# Alendronate-functionalized double network hydrogel scaffolds for effective osteogenesis

**DOI:** 10.3389/fchem.2022.977419

**Published:** 2022-08-17

**Authors:** Guoke Tang, Liang Zhu, Weiheng Wang, Dongqing Zuo, Changgui Shi, Xiaojie Yu, Rui Chen

**Affiliations:** ^1^ Department of Orthopedics, Second Affiliated Hospital of Naval Medical University, Shanghai, China; ^2^ Department of Orthopedics, Shanghai General Hospital, Shanghai Jiaotong University, Shanghai, China; ^3^ Department of Orthopedics, Hunan Aerospace Hospital, Changsha, Hunan, China

**Keywords:** GelMA, ALN, OSA, schiff base, DN hydrogel, osteogenic differentiation

## Abstract

Development of artificial bone substitutes mimicking the extracellular matrix is a promising strategy for bone repair and regeneration. In views of the actual requirement of biomechanics, biodegradability, and bioactivity, herein, a double-network (DN) hydrogel was constructed by interspersing a methacrylated gelatin (GelMA) network into alendronate (ALN)-modified oxidized alginate (OSA) network via Schiff base reaction and photo-crosslinking process to promote *in situ* bone regeneration. This GelMA@OSA-ALN DN hydrogel possessed favorable network and pores, good biocompatibility, and enhanced biomechanics. Notably, the introduction of Schiff base furnished the ND hydrogel scaffold with pH-responsive biodegradation and sustained ALN drug release delivery, which could provide effective bioactivity, upregulate osteogenesis-related genes, and promote the cell viability, growth, proliferation, and osteogenesis differentiation for bone regeneration. Therefore, we provide a new insight to develop functional DN hydrogel scaffold toward governing the on-demand drug release and achieving the stem cell therapy, which will be developed into the minimally invasive gelling system to prolong local delivery of bisphosphonates for the bone-related diseases.

## Introduction

Natural bone tissue, as a complex material, consists of many typical inorganic and organic composites with elaborately hierarchical architectures at macro-, micro- and nano-scales and high mechanical performance (Dimitriou et al., 2011; Almubarak et al., 2016). However, bone defect has developed as one of the most common clinical symptoms until now, which is generally induced by repulsive bone infection, tumor, congenital diseases, and accidental traumatic events (Thomas and Puleo, 2011; Oryan et al., 2014; Miller and Chiodo, 2016; Zeng et al., 2018). Bisphosphonates, due to their remarkable affinity and high selectivity to bone minerals, can prevent bone loss in patients, and are widely used in the therapy of many bone-related disease. Importantly, bisphosphonates were reported to have the capacity to improve the osteogenic differentiation of stem cells via elevating osteogenic gene expression (Loessner et al., 2016; Byambaa et al., 2017; Shi et al., 2017; Liu et al., 2018). As a typical representative of bone resorption inhibitor, alendronate (ALN) is a widely used oral amino-bisphosphonate drug thats inhibits resorptive activity, but the extraordinary hydrophilicity can generally result in low oral bioavailability and gastrointestinal permeability (Wei et al., 2019; Li et al., 2020; Shi et al., 2022). Therefore, pursuing and developing the intelligent drug vector has been a promising direction for treatment of bone-related diseases.

In the past few decades, artificial substitutes have become preferred choices because of their wide source and good biocompatibility. Biomedical materials are a series of engineering scaffolds that can simulate the composition and structure of native bone tissue and facilitate the cell growth and differentiation at the defect site, thereby promoting the bone tissue regeneration (Sun et al., 2012; Wang et al., 2020; Tang et al., 2021; Zhu et al., 2022). Among them, naturally polymeric hydrogel is a promising biomaterial with the 3D crosslinking networks, similar native extracellular matrix, uniquely physical properties, and flexible chemical modification, which has been recognized as an ideal tissue engineering scaffold because of its biocompatibility, easy accessibility, renewable sources and *in situ* formability (Wang et al., 2019; Tang et al., 2020; Liu et al., 2021; Zhang et al., 2022a; Zhang et al., 2022b; Peers et al., 2022; Yang et al., 2022). Wherein, gelatin methacryloyl (GelMA) as a derivative of gelatin with native Arg-GlyAsp (RGD) sequences is a kind of denatured products of collagen. It possessed good biocompatibility, easy gelation, low antigenicity, and biological function that can promote cell adhesion, proliferation, and differentiation for engineered bone scaffolds both *in vitro* and *in vivo* (Yue et al., 2015; Jia et al., 2016). In addition, it also has been made great progress with multitudinous fields ranging from tissue engineering to drug delivery; however, its poor mechanics and uncontrollable degradation behavior greatly limit its medical application, especially for bone defect repair (Billiet et al., 2014; Moreira et al., 2019; Zhang et al., 2019; Li et al., 2022). Chemical immobilization and controlled release of ALN drugs in hydrogel materials is an interesting area of research with special emphasis on bone regeneration and treatment of bone diseases.

To solve this problem, scientists have tried to introduce other polymeric moieties to improve the properties through many approaches and strategies. As a representative, sodium alginate (SA), due to the good biocompatibility, low toxicity, non-immunogenicity, easy accessibility, and renewable sources, is widely used in tissue engineering, food, and cosmetics (Liu et al., 2020; Varaprasad et al., 2020; Zheng et al., 2021). However, its large number of carboxyl and hydroxyl groups make the hydrophilic SA degrade uncontrollably and swell massively, and therefore the poor stability significantly restricts its practical applications (Hernández-González et al., 2020; Montes et al., 2022; Wang et al., 2022). Therefore, introduction of oxidized sodium alginate (OSA) into the GelMA through the Schiff base reaction between the aldehyde groups and amino groups is an optimal choice to construct the DN hydrogels with excellent biocompatibility, tailored biodegradability and high mechanical properties. These phosphonate-containing polymers have already been used for the clinical treatment of osteoporosis and other similar diseases, which can be a promising candidate for novel biomaterial scaffolds to promote osteogenesis and biomineralization.

Here in this work, integrated with simultaneous requirement of preferred scaffold and smart drug formulation, we designed and prepared a drug-loaded scaffold of GelMA-OSA@ALN DN hydrogel for bone repair. By means of the Schiff base reaction and photopolymerization in aqueous solution, the GelMA-OSA@ALN DN hydrogel was fabricated with naturally analogue extracellular matrix, suitable network structures and preferable mechanical properties, which provided more chance on promoting the viability, growth and proliferation of bone marrow derived mesenchymal stem cells (BMMSCs). Due to the connected Schiff base linkages among the network backbone and ALN drugs, this DN hydrogel scaffold displayed a pH-responsive degradation and a sustained drug release, thereby allowing the adaptation of hydrogels to promote osteogenic differentiation and bone regeneration with high therapeutic efficiency. Consequently, we believe this work is of great benefit to broaden the perception to construct more qualified engineering substitutes and offer a general strategy on governing the on-demand drug release for tissue repair.

## Materials and methods

### Materials

Gelatin (80–100 kDa, J&K), sodium alginate (SA, 98%, J&K), methacrylate anhydride (98%, J&K), sodium periodate (NaIO_4_, Aladdin), alendronate (ALN, 99%, Energy Chemical). All other reagents were analytical grade and used as received without further purification.

### Measurements

The ^1^H NMR spectroscopy were applied on a Bruker DRX-400 spectrometer using D_2_O with concentration of 10 mg/ml. Scanning electron microscopy (SEM) images were obtained on a JSM-6700F microscope (JEOL, Japan), and the morphologies of the hydrogel surfaces were observed at an accelerating voltage of 10 kV. The freeze-dried samples were sputter-coated with a thin layer of Pt for 90 s to make the conductive sample before testing. The compressive profiles of GelMA@OSA and GelMA@OSA-ALN hydrogels were measured with a beam velocity of 1 mm/min using a testing machine of Instron 3,365. The diameter and thickness of cylindrical samples were 10 mm and 5 mm, respectively. Five samples were measured for each group. Confocal laser scanning microscopy (CLSM) image was obtained on a Zeiss LSM 510 microscope. The ALN release was measured by high performance liquid chromatography (HPLC) on a Shimadzu LC-20AT system with UV detection at 266 nm.

### Synthesis of GelMA

GelMA was prepared according to the previous literature (Liu et al., 2018). Briefly, 20 g of gelatin was fully dissolved in 150 ml of distilled water at 50 C. Then, 30 ml of methacrylic anhydride was added into the solutions under vigorous stirring for 4 h. After that, the impurities were removed by the dialysis (MW cutoff, 3,500 Da) against deionized water for 4 days and collected after freeze-drying to afford the white powder of GelMA under vacuum.

### Synthesis of OSA

Oxidized sodium alginate was prepared according to the previous literature (Wang et al., 2022). Briefly, 10 g of SA was fully dissolved in 200 ml of distilled water in a dark bottle, and then 50 ml of anhydrous ethanol was added into the solutions under vigorous stirring. After that, a certain amount of NaIO_4_ was added into solutions at 25 C in N_2_ atmosphere. After stirring for a period of 12 h, 2 ml of ethylene glycol was incorporated to reduce the unreacted NaIO_4_. Subsequently, an amount of anhydrous ethanol and 5 g of NaCl were added to precipitate OSA, and the impurities were removed by the dialysis (MW cutoff, 3,500 Da) against deionized water for 4 days and collected after freeze-drying to afford the white powder of OSA under vacuum.

### Synthesis of OSA-ALN

Briefly, 1 g of OSA and a certain amount of ALN drug were dissolved in 20 ml of aqueous solutions under vigorous stirring for 24 h. After that, the impurities and unreacted byproducts were removed by the dialysis (MW cutoff, 1,000 Da) against deionized water for 4 days and collected after freeze-drying to afford the powder of OSA-ALN under vacuum.

### Preparation of GelMA@OSA and GelMA@OSA-ALN hydrogels

The hydrogels were simply prepared by adding the stock solution of OSA or OSA-ALN (3 wt%, 1 ml) to the stock solution of GelMA (15 wt%, 1 ml) containing the photoinitiator for 6 h followed by the UV irradiation at room temperature.

### *In vitro* drug release from the hydrogel

The hydrogel sample was prepared in a container with a diameter of 10 mm and height of 2 mm, and the GelMA@OSA-ALN hydrogel was immersed into various PBS solutions (pH 7.4 and 6.5), which were then collected at special intervals of time to measure the ALN drug concentration by HPLC.

### *In vitro* cytotoxicity assay

The cell viability was conducted by CCK-8 cytotoxicity assay. The cells were suspended in culture medium and seeded into 48-well plates with a density of 1 cells/100 µL × 10^4^ cells/100 µL in each well and incubated for 24 h at 37°C in a 5% CO_2_ humidified incubator. The hydrogels were immersed in the fresh cell medium for 24 h to get the extracts, and then the treated cell medium was used to replace the fresh cell medium to further incubate cells for another 24 h. The cells cultured in fresh medium were used as control. The cell number was correlated with optical density (OD).

### Cell proliferation

Cells were seeded and incubated in growth medium for 24 h, extracted hydrogel sample was added and incubated for another 24 h. After 7 days of culture, the cell culture medium was removed and then 100 µL of fresh culture medium and 10 µL of CCK-8 were added to the 96 wells for 4 h. Finally, the absorbance was read at 450 nm on a microplate reader.

### Live/dead staining assay

Cell live and dead viability was determined by Live/dead viability. The staining reagent mixture including the red fluorescent propidium iodide (PI) stain and green fluorescent (AM) stain were added to the reaction mixture and incubated in the dark at a room temperature for 15 min. The corresponding fluorescence emission was assessed using CLSM.

### Osteogenesis-related gene expression

The expression of osteogenesis-related marker genes (Runx 2, ALP, COL-1, OCN and OPN) was detected by real-time PCR. The total RNA was extracted using TRIzol Reagent and cDNA was prepared from 200 ng of total RNA by RevertAidTM H Minus First Strand cDNA Synthesis Kit. Relative quantification of target genes was normalized to internal reference.

### Statistics analysis

All results were presented as mean and standard deviation with 3-6 independent experiments. Statistics were analyzed using the SPSS software. When *p* < 0.05, differences were significant.

## Results and discussion

### Preparation and characterization of GelMA@OSA-ALN DN hydrogel.

**FIGURE 1:**
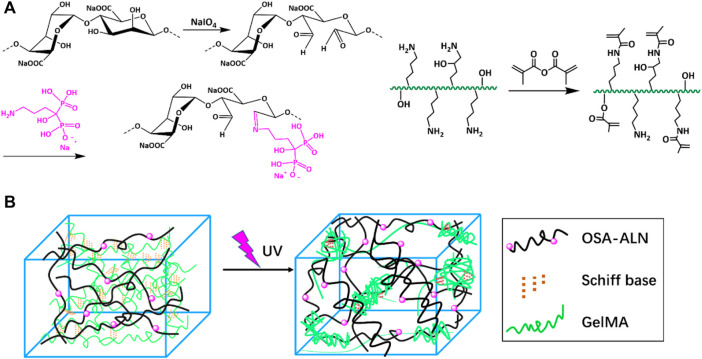
Schematic illustration. **(A)** Synthesis route of the OSA-ALN polymer, GelMA polymer and **(B)** the preparation of GelMA@OSA-ALN hydrogel.

**FIGURE 2:**
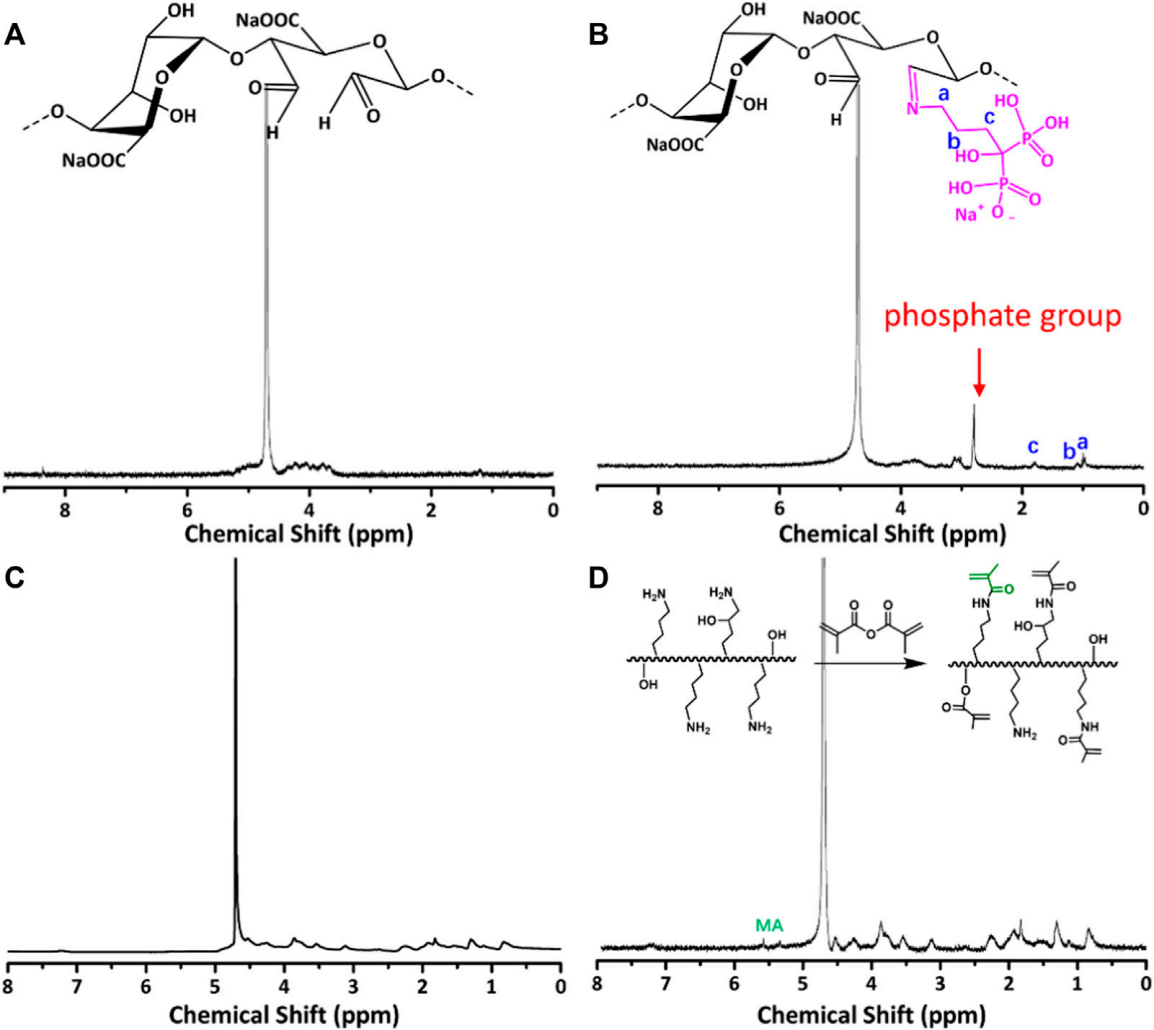
^1^H NMR spectra of **(A)** OSA, **(B)** OSA-ALN, **(C)** Gel and **(D)** GelMA polymers.

The synthetic route of GelMA@OSA-ALN DN hydrogel was clearly demonstrated in Figure 1. On account of many hydrophilic groups (-OH, -COOH) on the backbone of alginate molecules, they can easily form intramolecular hydrogen bonds with rigid molecular structure of alginate. However, the inert dihydroxy groups on the alginate backbone can be oxidized to generate the reactive dialdehyde groups upon exposure to oxidative NaIO_4_, which made the cleavage of the C2–C3 bond of the alginate uronic acid monomer to obtain the OSA polymer. Then, the small ALN molecules can be chemically linked through the quick Schiff base reaction in mild conditions while the GelMA macromolecule was easily prepared using the high-efficient reaction in aqueous solutions. As shown in Figure 2A, the proton peaks of SA appearing at 3.7∼3.55 ppm that were assigned to the H2 of α-L-guluronic acid decreased and moved to the high field for cleavage of the C2–C3 bond of the uronic acid monomer. The appearance of these new proton signal peaks and the change of proton signal peaks also directly proved the successful oxidation of alginate with NaIO_4_ to obtain the OSA polymer. In addition, the typical characteristic peaks (a, b, c) in Figure 2B were attributed to the ALN molecules, verifying the successful modification to obtain the targeted OSA-ALN. Figures 2C,D showed that the proton peaks of the methyl groups located at 5.3 and 5.6 ppm verified the favoring introduction of double bonds into gelatin and the successful preparation of GelMA macromolecule. On account of the residual aldehyde group within the OSA or OSA-ALN, the terminated amine groups of GelMA can also be reacted with aldehyde group via the Schiff base reaction to form the first chemical crosslinking network. Upon the exposure to UV irradiation, the following polymerization could enhance the degree of crosslinking and form the chemical-chemical double network hydrogels. It is mentioned that the introduction of Schiff base bonds furnished the GelMA@OSA-ALN DN hydrogels with pH-responsive drug release as well as the controllably biodegradable behaviors. During this gelling process, the adhesion strength onto the tissue could be meanwhile achieved via the formation of Schiff base linkages among the amine groups of protein tissues and aldehyde groups of OSA polymer, demonstrating its facile application in the bone-related disease.

**FIGURE 3:**
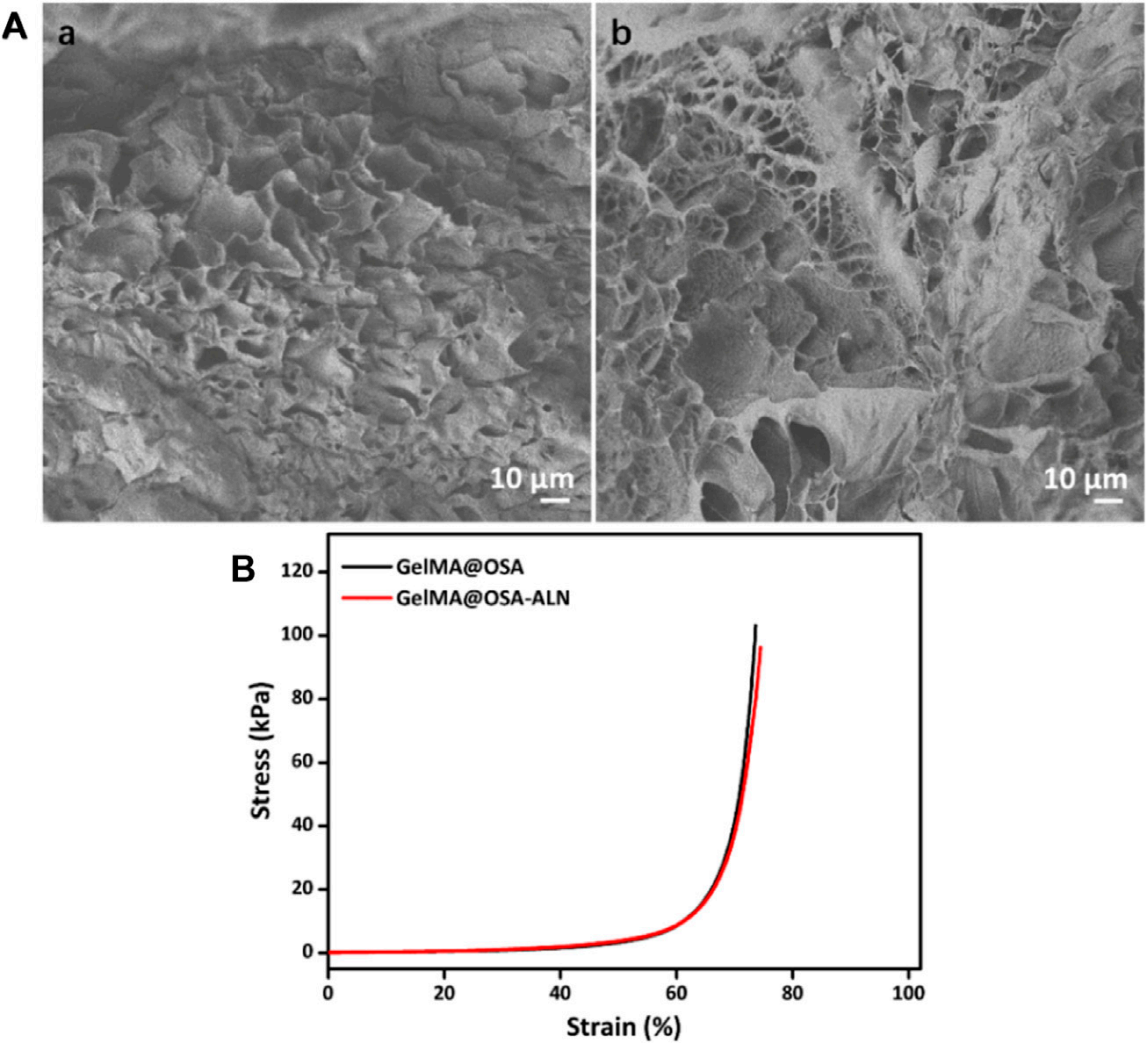
Morphological and mechanical characterizations. **(A)** SEM images and **(B)** Compressive curves of **(a)** GelMA@OSA and **(b)** GelMA@OSA-ALN DN hydrogels.

The morphologies of GelMA@OSA and GelMA@OSA-ALN DN hydrogels were observed by SEM images in Figure 3A, which showed the similar structures of GelMA@OSA and GelMA@OSA-ALN DN hydrogels without significant difference. These hydrogel scaffolds possessed large pore size and uneven porosity that could satisfy host cell entry and nutrition and metabolic waste exchange. Understanding this circumstance, GelMA@OSA-ALN DN hydrogel could enable the sustained release of internal ALN drugs, stem cell infiltration, and intra-extra substance exchange from the hydrogel scaffolds. The compressive stress and modulus were important parameters to assess the mechanical stability of the DN hydrogel scaffolds. As shown in Figure 3B, these GelMA@OSA and GelMA@OSA-ALN DN hydrogels also exhibited approximate compressive behaviors with the analogous strength and modulus, further revealing the introduction of small amount of ALN didn’t affect the uniform network and the mechanical strength for the DN hydrogels. It was mentioned that the lower compression deformation behaviors may be ascribed to their rigid backbone architectures and dense crosslinking network of the DN hydrogels.

### *In vitro* drug release

**FIGURE 4:**
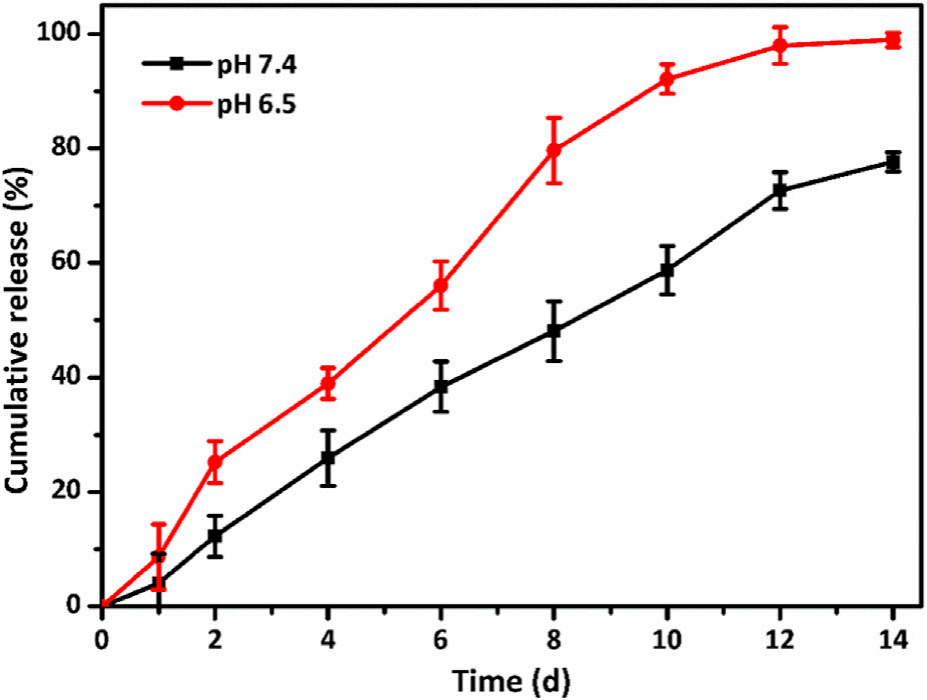
Drug release profiles. *In vitro* ALN release behavior from the GelMA@OSA-ALN DN hydrogels at various (pH 7.4 and 6.5) PBS solutions.

The release profile of ALN laden in the GelMA@OSA-ALN DN hydrogel *in vitro* was assessed in Figure 4. Since the Schiff base bonds were distributed within the GelMA@OSA-ALN DN hydrogels, there is an obvious pH-triggered drug release behavior in acidic conditions compared to the pH 7.4 solutions. On account of the neutral or weak acidic environment during the early phase of bone regeneration, it was necessary to assess the ALN drug release in these two different conditions. As shown in Figure 4, a sustained release of ALN drug was observed for more than 2 weeks in both pH solutions. The obvious drug release rate revealed the breakage of Schiff base bonds and gradual degradation of GelMA@OSA-ALN DN hydrogel at pH 6.5 solutions. With the extension of time, a cumulative burst release of ALN drug quickly reached round 38.6% at day 4 and gradually approached its plateaus with nearly 97% at day 12, suggesting the Schiff base breakage and correspondingly sustained original ALN release into the solutions. In contrast, GelMA@OSA-ALN DN hydrogel could also perform a gradient release behavior with the release content of more than 70% at day 14 under the slow hydrolysis effects, which indicates its superior capacity on maintaining high drug concentration to sustainedly activate the repair mechanism in the defect area and achieving the therapeutic effect for a long period of time.

### Cell viability and proliferation

**FIGURE 5:**
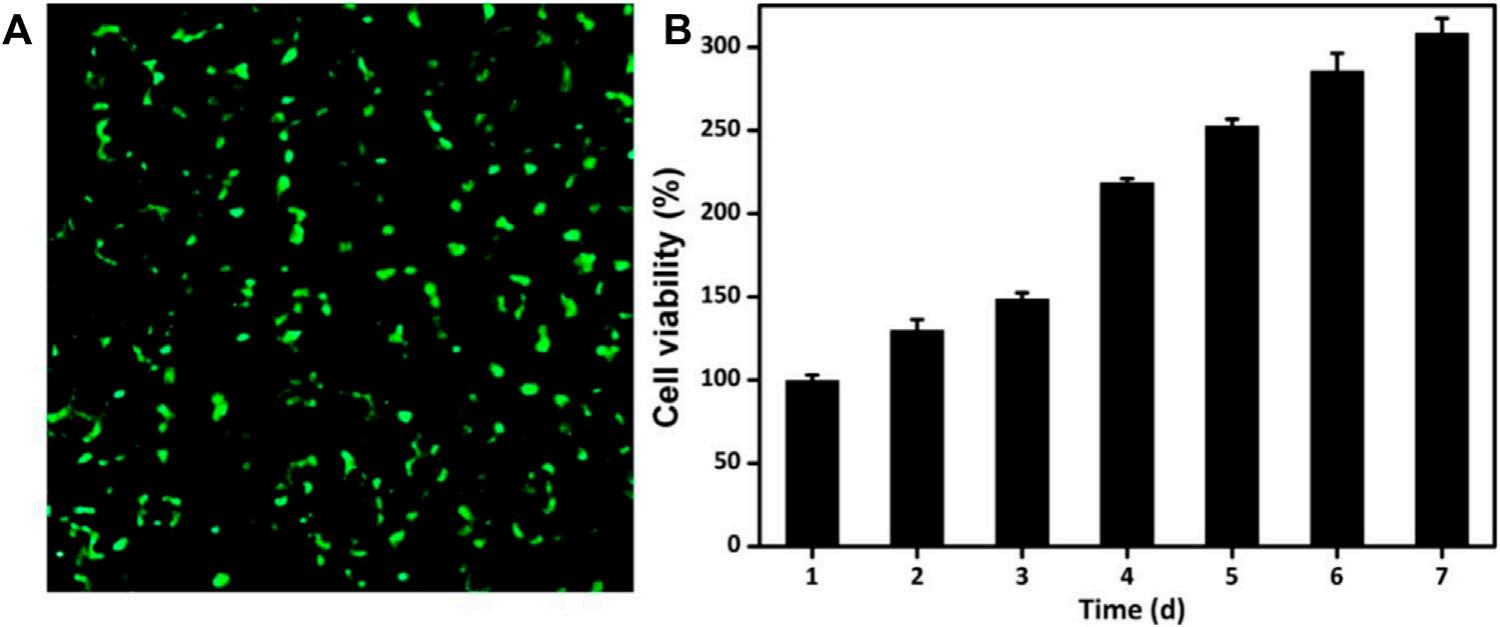
Cytotoxicity of GelMA@OSA-ALN DN hydrogels *in vitro*. **(A)** CLSM images of the cells stained with a live/dead reagent on 24 h. The live and dead cells were stained green and red, respectively. **(B)** Cell proliferation was detected after 7 days of culture.

The ability to maintain cell activity and support cell proliferation is significantly important for the drug-loaded engineering scaffolds. GelMA and OSA polymer are recognized as nontoxic and used extensively biomaterials in engineering applications and regenerative medicine. To evaluate the cytocompatibility of GelMA@OSA-ALN DN hydrogel, live/dead staining and CCK-8 assays were performed in Figure 5A. The luminous green fluorescence indicated its biocompatibility after *in vitro* culture for 24 h. In addition, the cells could proliferate well in this DN hydrogel in the long-term because the cell numbers showed a clear increase from day 1 to day 7 in Figure 5B. Cell proliferation rate was slightly increased in the initial 3 days after co-culturing and reached a significant increase in the following 4 days of culture *in vitro*, which demonstrated its high cell survival, growth and proliferation rates that can be regarded as a preliminary indication of implanted DN hydrogel scaffolds in the biomedical applications.

### Osteogenic differentiation of hydrogel scaffolds *in vitro*

**FIGURE 6:**
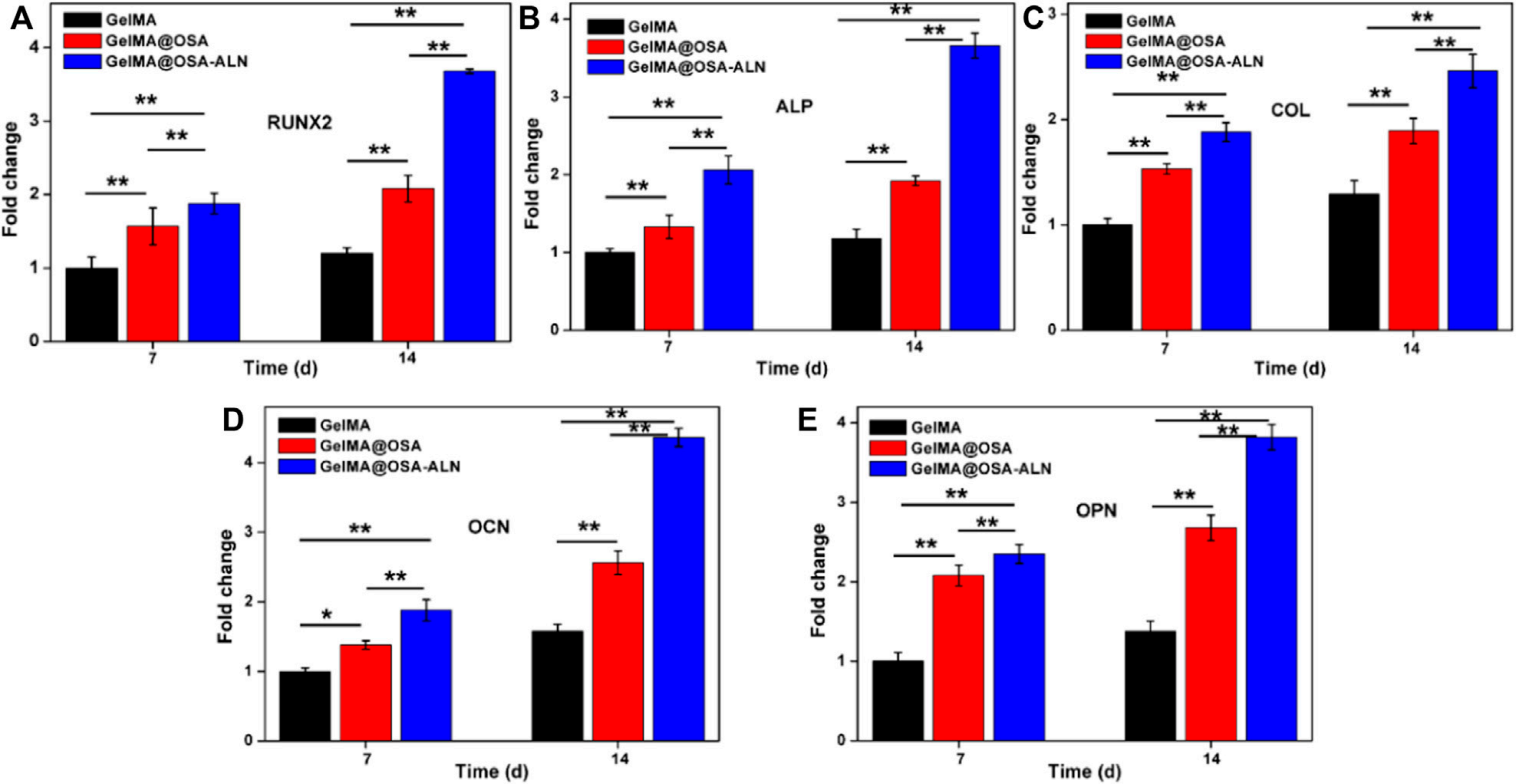
qRT-PCR analysis of mRNA levels of osteogenic genes. Relative gene expression of **(A)** Runx 2, **(B)** ALP, **(C)** COL, **(D)** OCN and **(E)** OPN on day 7 and 14 of culture. Statistically significant differences of GelMA, GelMA@OSA and GelMA@OSA-ALN (^∗^ *p* < 0.05, ^∗∗^ *p* < 0.01).

Ideal engineering bone repair scaffolds should enhance the osteogenic differentiation. To assess the functional effects of alendronate modification and mechanical effect of hydrogel scaffolds on the osteogenic differentiation in the cells, the mRNA levels of the expression of key marker genes Runx 2, ALP, COL, OCN and OPN were investigated using the GelMA, GelMA@OSA, and GelMA@OSA-ALN hydrogels in Figure 6. At day 7, real-time PCR showed that all the five key osteogenic markers in the GelMA@OSA-ALN group were significantly upregulated compared to other groups. It was mentioned that the higher expression in the GelMA@OSA hydrogel compared to the GelMA hydrogel indicated high mechanical strength and promoted osteogenic differentiation ability due to the introduction and contribution of alginate moieties. At day 14, these levels of GelMA@OSA-ALN group were also significantly higher than those in the other groups, suggesting that the released ALN drugs could effectively promote osteogenic differentiation for a long period of time *in vitro*, which was consistent with the long-term sustained drug release behavior (Figure 4). Furthermore, this sustainable ALN release and high therapeutic ossification effect also testified that bisphosphonates can indeed promote the expression levels of osteogenic-related genes in osteoblasts and improve osteogenic differentiation of stem cells. It was mentioned that low-dose of alendronate (<1.5 mmol) can serve as an optimal osteo-inductive factor to promote osteogenesis *in vitro* and accelerate the osteoblastic differentiation of cells for bone regeneration *in vivo* (Wang et al., 2009; Wang et al., 2010; Chang et al., 2022). Therefore, we have directly used the low drug concentration of ca. 1 mmol and not explored the effect of ALN concentration on the osteogenesis.

## Conclusion

In summary, we developed a biocompatible and biodegradable GelMA@OSA-ALN DN hydrogel with the preferable biomechanics and bioactivity for effective osteogenesis. Its biofriendly components, porous architectures, and satisfactory mechanical performance could facilitate the cell viability, growth, and proliferation. In addition, the pervasive Schiff-base within the hydrogels enabled the drug-loaded DN hydrogel to adapt the neutral or weak acidic environment during the early phase of bone regeneration, thus achieving sustained drug release behavior for more than 2 weeks and promoting the therapeutic efficiency toward osteoblast differentiation *in vitro*. Consequently, the findings imply that these DN hydrogels mediate optimal release of therapeutic cargoes and promote in bone regeneration, which will be broadly utilized in tissue engineering and regenerative medicine.

## Data Availability

The raw data supporting the conclusions of this article will be made available by the authors, without undue reservation.
